# Choroidal Osteoma: Case Series

**DOI:** 10.1155/carm/8005092

**Published:** 2026-07-18

**Authors:** Alua Aubakirova, Kamilya Sarsembekova, Zaure Jumatayeva, Zauresh Utelbayeva, Zhanerke Serikova, Dinara Berlibek

**Affiliations:** ^1^ 3^rd^ Ophthalmological Department, Kazakh Eye Research Institute, Almaty, Kazakhstan; ^2^ Clinical-Diagnostic Department, Kazakh Eye Research Institute, Almaty, Kazakhstan; ^3^ Department of Ophthalmology, Asfendiyarov Kazakh National Medical University, Almaty, Kazakhstan, kaznmu.kz

**Keywords:** choroidal neovascularization, choroidal osteoma, intraocular tumor, multimodal imaging, optical coherence tomography

## Abstract

**Purpose:**

To describe the clinical presentation, multimodal imaging findings, and management of patients with choroidal osteoma observed at a tertiary ophthalmic center.

**Methods:**

A retrospective case series of three patients (four affected eyes) diagnosed with choroidal osteoma at the Kazakh Eye Research Institute, Almaty, Kazakhstan. All patients underwent a comprehensive ophthalmic examination, including best‐corrected visual acuity assessment, fundus photography, B‐scan ultrasonography, optical coherence tomography (OCT), fluorescein angiography (FA), OCT angiography, and computed tomography (CT).

**Results:**

The patients presented with decreased vision, progressive visual impairment, or vitreous floaters. Choroidal osteoma was identified as a yellowish‐white or orange peripapillary subretinal lesion with well‐defined borders. Multimodal imaging demonstrated characteristic calcified choroidal lesions with high acoustic reflectivity on ultrasonography, hyperdense plaques on CT, and hyperreflective heterogeneous structures on OCT corresponding to cancellous bone architecture. None of the patients demonstrated active choroidal neovascularization at presentation. Associated systemic or inflammatory conditions included systemic lupus erythematosus, retinal vasculitis, and chronic infectious exposure. All patients were managed conservatively with regular follow‐up because of the absence of severe complications requiring intervention.

**Conclusions:**

Choroidal osteoma is a rare benign ossifying tumor that predominantly affects young individuals and may be associated with inflammatory or immune‐mediated conditions. Multimodal imaging is essential for accurate diagnosis and monitoring. Although tumor progression is usually slow, long‐term surveillance is necessary because of the risk of decalcification, retinal pigment epithelium atrophy, serous retinal detachment, and choroidal neovascularization.

## 1. Introduction

Choroidal osteoma (CO) is a rare benign ossifying tumor characterized by the presence of mature cancellous bone within the choroid [1]. The condition was first described by Gass in 1978 as a distinct choroidal tumor associated with visual disturbances [2]. Although choroidal osteoma is considered an uncommon ocular tumor, its incidence remains unknown.

Choroidal osteoma has been reported in individuals of all ethnic groups and most commonly affects young women in the second to third decades of life. Approximately 80% of cases are unilateral [1]. Patients may present with blurred vision, metamorphopsia, photophobia, decreased visual acuity, or visual field defects; however, the disease may remain asymptomatic in 8%–30% of cases [3, 4].

Choroidal osteoma typically appears as a slightly elevated yellow‐orange or yellow‐white subretinal lesion with well‐defined geographic or scalloped borders, often located in the peripapillary or macular zone [5, 6]. Morphologically, CO demonstrates mature bony trabeculae with marrow‐like spaces containing dilated thin blood vessels, commonly referred to as “spider vessels,” which are believed to connect the choriocapillaris with larger choroidal vessels [2].

In this report, we present a series of three clinical cases of choroidal osteoma evaluated using multimodal imaging techniques.

## 2. Clinical Case No. 1

A 20‐year‐old female patient presented with decreased vision in both eyes. Her medical history was significant for systemic lupus erythematosus (SLE), diagnosed 6 years earlier. The patient had been treated with hydroxychloroquine for 5 years but had discontinued therapy 6 months before presentation.

Objective findings are as follows:

VIS OD = 0.08, BCVA = 0.8.

VIS OS = 0.1, BCVA = 1.0.

Intraocular pressure (IOP) = 20/19 mmHg.

**FIGURE 1:**
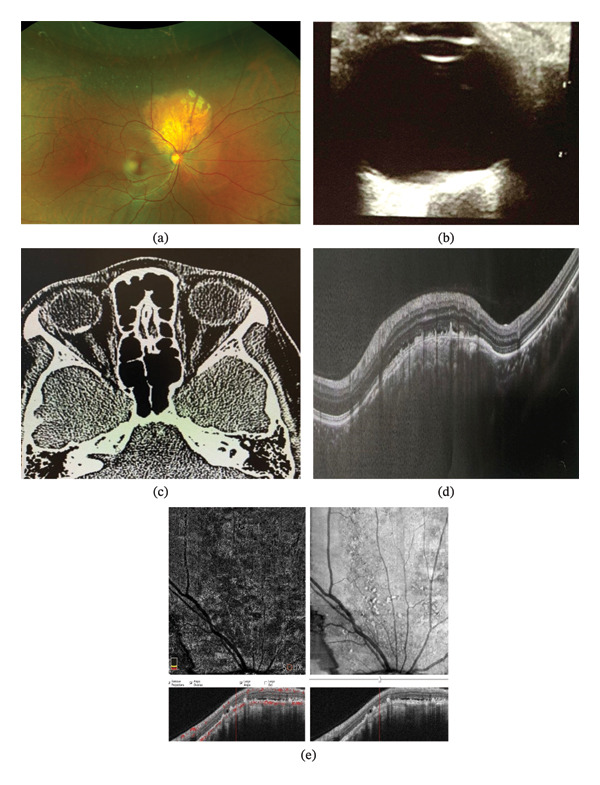
Clinical Case 1: (a) fundus photography—white‐yellowish chorioretinal lesion with patchy pigment deposits, (b) B‐scan ultrasound of the right eye—a hyperechogenic prominent lesion in the posterior pole of the eye globe, (c) CT scan of the right orbit—a plaque‐like intraocular lesion with bone‐equivalent density in the choroid, (d) OCT of the right eye—retinal prominence above the optic nerve head due to a hyperreflective lesion, (e) OCT angiography—subretinal lesion with multiple hyperreflective foci (calcifications) within different retinal layers and shallow neuroepithelial detachment without choroidal neovascularization.

Fundus examination of the right eye revealed a large yellow‐white chorioretinal lesion with patchy pigment deposits located superior to the optic disc (Figure 1a).

B‐scan ultrasonography demonstrated a hyperechogenic lesion in the posterior pole measuring 2.44 mm in thickness and 8.33 mm in basal diameter, with posterior acoustic enhancement. Visualization of intrinsic vascularity was limited because of the relatively small lesion size (Figure 1b).

Computed tomography (CT) of the orbit confirmed the diagnosis of choroidal osteoma by demonstrating a plaque‐like intraocular lesion with bone‐equivalent density in the choroid (Figure 1c).

Optical coherence tomography (OCT) showed retinal prominence above the optic nerve head area caused by a hyperreflective lesion at the level of the pigment epithelium with indistinct borders (Figure 1d). OCT angiography (OCTA) revealed a subretinal lesion with multiple hyperreflective foci (calcifications) within different retinal layers without choroidal neovascularization (CNV) (Figure 1e).

Due to the absence of complications such as retinal pigment epithelium (RPE) atrophy over the area of decalcified osteoma, serous retinal detachment caused by pigment epithelium decompensation, and CNV, dynamic monitoring every 6 months was recommended.

## 3. Clinical Case No. 2

A 16‐year‐old male patient presented with progressive visual loss, more pronounced in the left eye. Four years earlier, he had been diagnosed with retinal vasculitis associated with retinal hemorrhages. Subsequently, he developed macular edema, retinal hemorrhages, and fibrosis consistent with secondary maculopathy.

Initially, the patient received conservative resorptive therapy. Intravitreal anti‐VEGF treatment was recommended; however, the patient declined the proposed therapy.

Objective findings are as follows:

VIS OD = 0.1, BCVA = 0.8.

VIS OS = 0.05, BCVA = 0.08.

IOP = 14/15 mmHg.

**FIGURE 2:**
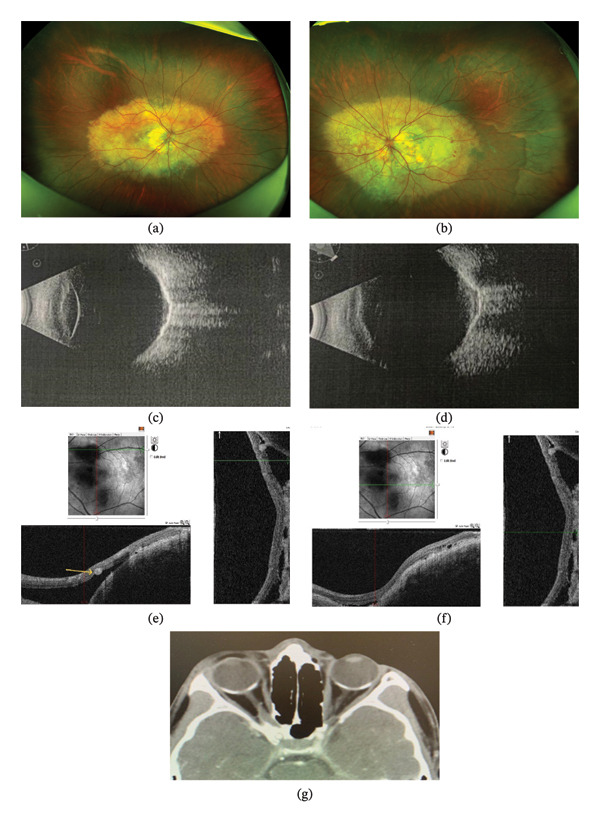
Clinical Case 2: (a, b) fundus photographs of both eyes—a peripapillary yellowish lesion with macular involvement, (c, d) B‐scan ultrasound of both eyes—increased echogenicity of the lesions is observed, OCT of the right eye, (e) the retinal profile is deformed by a prominent subretinal lesion with increased hyperreflectivity at the retinal pigment epithelium—Bruch’s membrane complex. A localized microcystic intraretinal edema is visualized over the area of prominence, (f) a hyperreflective lesion corresponding to a calcification (indicated by the yellow arrow) is observed extending from the outer plexiform layer to the ellipsoid zone of the neuroepithelium, (g) CT scan of the orbits of both eyes—CT signs of calcification in the posterior poles of both eyes.

Fundus examination demonstrated bilateral peripapillary yellowish lesions with well‐defined borders. Fibrotic retinal changes and retinal hemorrhages were observed in the left eye (Figure 2a,b).

B‐scan ultrasonography demonstrated vitreous opacities in both eyes, increased echogenicity of the posterior pole lesions, irregular ocular contour, and posterior acoustic enhancement (Figure 2c,d).

OCT showed an elevated subretinal lesion deforming the retinal contour, along with focal intraretinal cystic edema. Multiple hyperreflective foci corresponding to calcifications were identified within the retinal layers (Figure 2e,f).

Orbital CT revealed bilateral calcified choroidal lesions without pathological contrast enhancement (Figure 2g).

The patient was advised to undergo regular follow‐up examinations every 6 months.

## 4. Clinical Case No. 3

A 28‐year‐old male patient presented with complaints of a persistent floater in the left eye that had been present for approximately 1 year.

Objective findings are as follows:

VIS OD = 0.2, BCVA = 1.0.

VIS OS = 0.2, BCVA = 0.9.

IOP = 21/21 mmHg.

**FIGURE 3:**
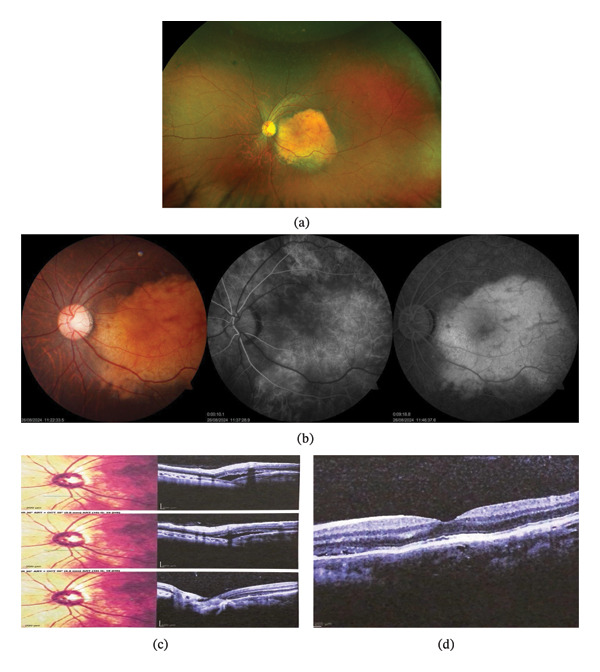
Clinical Case 3: (a) Left eye fundus image—yellowish subretinal lesion in the posterior pole, (b) fluorescein angiography of the left eye—early hyperfluorescence during the arterial phase with progressive hyperfluorescence in subsequent phases and persistent late staining, (c, d) OCT of the left eye demonstrating deformation of the macular contour, neuroepithelial detachment, intraretinal cystic spaces, isolated hyperreflective foci of destruction, a prominent subretinal hyperreflective lesion, and alteration of the choroidal profile.

Fundus examination of the left eye revealed a yellowish subretinal lesion with well‐defined borders located in the posterior pole (Figure 3a).

Laboratory testing revealed IgG antibodies against Opisthorchis, Listeria, cytomegalovirus, and herpes simplex virus.

Fluorescein angiography revealed a pigmented lesion with distinct borders in the macular area. The lesion demonstrated early hyperfluorescence during the arterial phase with progressive hyperfluorescence in subsequent phases and persistent late staining. Intrinsic tumor vessels were not visualized (Figure 3b).

On OCT of the left eye (Figure 3c,d), the macular contour was deformed, with neuroepithelial detachment, intraretinal cystic spaces, isolated hyperreflective foci of destruction, a subretinally prominent hyperreflective lesion, and deformation of the choroidal profile.

The patient was referred for treatment to an infectious disease specialist and recommended dynamic monitoring every 6 months.

## 5. Discussion

The etiology and pathogenesis of choroidal osteoma remain incompletely understood. Several mechanisms have been proposed, including inflammatory, hormonal, metabolic, hereditary, and developmental factors; however, none has been conclusively established as the primary cause of the disease. The hypothesis that choroidal osteoma represents a choristoma is supported by the presence of mature osseous tissue within the choroid, although this theory does not fully explain the female predominance or the progressive enlargement of lesions during adulthood [7]. Likewise, no consistent association has been demonstrated between choroidal osteoma and systemic calcium, phosphate, or alkaline phosphatase abnormalities.

Recent reports have suggested a possible relationship between chronic inflammation and the development of choroidal osteoma. Associations between central serous chorioretinopathy, inflammatory retinal disorders, and chronic infectious conditions have been described in the literature. Proposed mechanisms include osteogenic differentiation stimulated by bone morphogenetic proteins (BMP‐7), transforming growth factor beta‐1 (TGF‐β1), inflammatory cytokines, and RPE‐derived mesenchymal stem cell activity [8–10]. In the present case series, all patients had a history suggestive of chronic inflammatory or immune‐mediated processes, including SLE, retinal vasculitis, and chronic infectious exposure. Although causality cannot be established from isolated observations, these findings support the hypothesis that chronic inflammation may contribute to the pathogenesis or progression of choroidal osteoma.

CNV is one of the most vision‐threatening complications of choroidal osteoma and has been reported in approximately 31%–47% of patients during long‐term follow‐up [3, 4]. The development of CNV is believed to result from progressive decalcification with subsequent disruption of the RPE and Bruch’s membrane, facilitating the ingrowth of abnormal choroidal vessels [3]. Histopathological studies demonstrating osteoclast‐like cells within neovascular membranes and OCT findings showing CNV arising from the osteoma itself further support this mechanism [6, 11]. In our series, none of the patients demonstrated CNV at presentation, emphasizing the importance of regular monitoring even in asymptomatic or minimally symptomatic cases.

Tumor decalcification represents another important prognostic feature. Approximately half of choroidal osteomas undergo partial or complete decalcification over time, resulting in thinning of the overlying retina, RPE atrophy, photoreceptor loss, and progressive visual decline, particularly when the fovea is involved [3, 12, 13]. Advanced OCT imaging has improved the characterization of these structural changes, demonstrating preservation of retinal architecture over calcified regions and degeneration within decalcified areas [11, 13–15]. In our patients, the OCT findings corresponded closely with previously described imaging characteristics, including hyperreflective lesions with heterogeneous internal reflectivity and alterations of the outer retinal layers.

Multimodal imaging plays a central role in the diagnosis and follow‐up of choroidal osteoma. Ultrasonography remains a highly valuable diagnostic tool because of the characteristic high acoustic reflectivity and posterior acoustic shadowing associated with calcified lesions [7, 14]. CT confirms the diagnosis by demonstrating plaque‐like hyperdense lesions with bone‐equivalent density. OCT and OCTA provide detailed assessment of retinal integrity, subretinal fluid, and possible neovascularization, while fluorescein angiography helps identify leakage, RPE alterations, and occult CNV.

Currently, there is no standardized treatment for uncomplicated choroidal osteoma, and observation remains the preferred strategy in stable cases without CNV or progressive visual deterioration. Therapeutic interventions are primarily directed toward secondary complications such as CNV and serous retinal detachment. Previous studies have demonstrated variable outcomes with laser photocoagulation, transpupillary thermotherapy, and photodynamic therapy, particularly for extrafoveal lesions [4, 16–23]. Recently, anti‐VEGF therapy has become the mainstay of treatment for CNV associated with choroidal osteoma, with favorable anatomical and functional outcomes reported after intravitreal bevacizumab or ranibizumab injections [24–26].

The present study has several limitations. First, this is a small retrospective case series from a single tertiary referral center, which limits the generalizability of the findings. Second, long‐term follow‐up data were limited, preventing assessment of lesion progression, decalcification, or delayed development of CNV. Nevertheless, the study highlights the importance of multimodal imaging in establishing the diagnosis and monitoring patients with this rare ocular tumor.

## 6. Conclusion

Choroidal osteoma is a rare benign ossifying tumor that predominantly affects young patients and may present with nonspecific visual symptoms or remain asymptomatic for prolonged periods. Multimodal imaging, including ultrasonography, OCT, OCTA, fluorescein angiography, and CT, is essential for accurate diagnosis and longitudinal assessment.

The cases presented in this series demonstrate the variable clinical manifestations of choroidal osteoma and suggest a possible association with chronic inflammatory or immune‐mediated conditions. Although the disease typically progresses slowly, patients remain at risk of vision‐threatening complications such as RPE atrophy, serous retinal detachment, decalcification, and CNV.

Careful long‐term follow‐up is therefore mandatory, even in clinically stable patients without initial complications. Early detection of secondary changes using multimodal imaging may facilitate timely intervention and help preserve visual function. Further multicenter studies with larger cohorts and longer follow‐up periods are required to clarify the pathogenesis, natural history, and optimal management strategies for choroidal osteoma.

## Author Contributions

Alua Aubakirova and Kamilya Sarsembekova developed the study conception and design. Material preparation and data collection and analysis were performed by Zaure Jumatayeva, Zauresh Utelbayeva, Dinara Berlibek, and Zhanerke Serikova. The first draft of the manuscript was written by Alua Aubakirova, Kamilya Sarsembekova, Zauresh Utelbayeva, Zhanerke Serikova, and Dinara Berlibek. Zaure Jumatayeva and Zauresh Utelbayeva critically reviewed the manuscript, provided valuable input, and contributed to its writing. All authors commented on previous versions of the manuscript.

## Funding

No funding was received for this research.

## Disclosure

We confirm that this manuscript is original, has not been published previously, and is not currently under consideration for publication elsewhere. All authors have approved the manuscript and agree with its submission to the journal. All authors read and approved the final manuscript.

## Consent

The authors certify that written informed consent was obtained from all patients included in this report. The consent explicitly covered the use of their clinical data and images for publication purposes. Ethics approval (approval no. 6‐2025) was granted by the Expert Council of Kazakh Eye Research Institute.

## Conflicts of Interest

The authors declare no conflicts of interest.
